# *BRCA* mutations and survival in breast cancer: an updated systematic review and meta-analysis

**DOI:** 10.18632/oncotarget.12158

**Published:** 2016-09-21

**Authors:** Yaning Zhu, Jian Wu, Chengwan Zhang, Suan Sun, Jian Zhang, Wenjie Liu, Jian Huang, Zhihong Zhang

**Affiliations:** ^1^ Department of Pathology, Huai'an First People's Hospital, Nanjing Medical University, Huai'an, Jiangsu Province, China; ^2^ The Central Laboratory of Huai'an First People's Hospital, Nanjing Medical University, Huai'an, Jiangsu Province, China; ^3^ Department of Breast Surgery, Huai'an First People's Hospital, Nanjing Medical University, Huai'an, Jiangsu Province, China; ^4^ Department of Pathology, the First Affiliated Hospital of Nanjing Medical University, Nanjing, Jiangsu Province, China

**Keywords:** breast cancer, BRCA mutation, survival, systematic review, meta-analysis

## Abstract

*BRCA* mutations occur frequently in breast cancer (BC), but their prognostic impact on outcomes of BC has not been determined. We conducted an updated meta-analysis on the association between *BRCA* mutations and survival in patients with BC. Electronic databases were searched. The primary outcome measure was overall survival (OS), and the secondary outcome measures included breast cancer-specific survival (BCSS) and event-free survival (EFS). Hazard ratios (HR) and 95% confidence interval (CI) were abstracted and pooled with random-effect modeling. Data from 297, 402 patients with BC were pooled from 34 studies. The median prevalence rates of *BRCA1* and *BRCA2* mutations were 14.5% and 8.3%, respectively. *BRCA* mutations were associated with worse OS (*BRCA1*: HR = 1.69, 95% CI, 1.35 to 2.12, *p* < 0.001; *BRCA2*: HR = 1.50, 95% CI 1.03 to 2.19, *p* = 0.034). However, this did not translate into poor BCSS (*BRCA1*: HR = 1.14, 95% CI, 0.81 to 1.16, *p* = 0.448; *BRCA2*: HR = 1.16; 95% CI 0.82 to 1.66, *p* = 0.401) or EFS (*BRCA1*: HR = 1.10, 95% CI, 0.86 to 1.41, *p* = 0.438; *BRCA2*: HR= 1.09; 95% CI 0.81 to 1.47, *p* = 0.558). Several studies analyzed *BRCA1* and *BRCA*2 mutations together and found no impact on OS (HR = 1.21; 95% CI, 0.73 to 2.00, *p* = 0.454) or EFS (HR = 0.94; 95% CI, 0.60 to 1.48, *p* = 0.787). *BRCA1* and *BRCA2* mutations were associated with poor OS in patients with BC, but had no significant impact on BCSS or EFS. An improved survival was observed in BC patients who had *BRCA1* mutation and treated with endocrinotherapy. The results may have therapeutic and prognostic implications important for *BRCA* mutation carriers with BC.

## INTRODUCTION

*BRCA1* and *BRCA2* are tumor suppressor genes identified in the early 1990s [1–4].The two genes are locate in chromosome 17q and 13q, respectively, and encode factors that inhibit cell growth. These factors are also involved in cell cycle control, gene transcription regulation, DNA damage repair, apoptosis and other important cellular processes. The common germline mutations of *BRCA1* are 5382 ins C, 185 del AG, 3819 del 5 and 4153 del A, while the common germline mutations of *BRCA2* include 4075 del GT and 5802 del4 [5]. Germline mutations of these genes confer an increased lifetime risk for a number of malignant tumors, especially breast cancer and ovarian cancer [6, 7]. Chen et al. reported that the cumulative risk for developing breast cancer ranged from 49% to 57% in women with *BRCA1* or *BRCA2* mutations by age 70 years [8].

Compared to non-carriers, *BRCA1*-associated breast cancers (BCs) are often high-grade and poorly differentiated infiltrating ductal carcinomas with special immunophenotypic features. These tumors are often triple negative ((estrogen receptor (ER), progesterone receptor (PR), and human epidermalgrowth factor receptor-2 (HER-2)) and express cytokeratins 5/6 (CK5/6), cyclin E and p53 [9–11]. However, it is controversial whether *BRCA* mutations in BC are associated with poor prognosis. Some studies demonstrated that *BRCA1/2* mutation carriers with breast cancer had a worse overall survival (OS) [12–22], others showed no significant difference when compared with non-carriers [23–41]. Some studies even showed *BRCA*-mutation carriers had better survival than non-carriers [42–44].

To address this uncertainty, two published meta-analyses have reported the effects of *BRCA1/BRCA2* mutations on BC survival [54, 56]. Lee et al. found that *BRCA1* but not *BRCA2* mutation decreased OS and PFS, while Zhong et al. suggested that *BRCA2* mutation was associated with worse OS, but not PFS, while *BRCA2* mutation was not associated with worse OS or PFS. We noted that these findings were limited by low statistical power.

Thus, we aimed to update the meta-analysis on the effect of *BRCA* mutation carriers versus non-carriers on survival in patients with BC, which may have a prognostic value in women with BC and an implication on genetic consoling for *BRCA* mutation carriers.

## RESULTS

### Literature search and study characteristics

**Figure 1:**
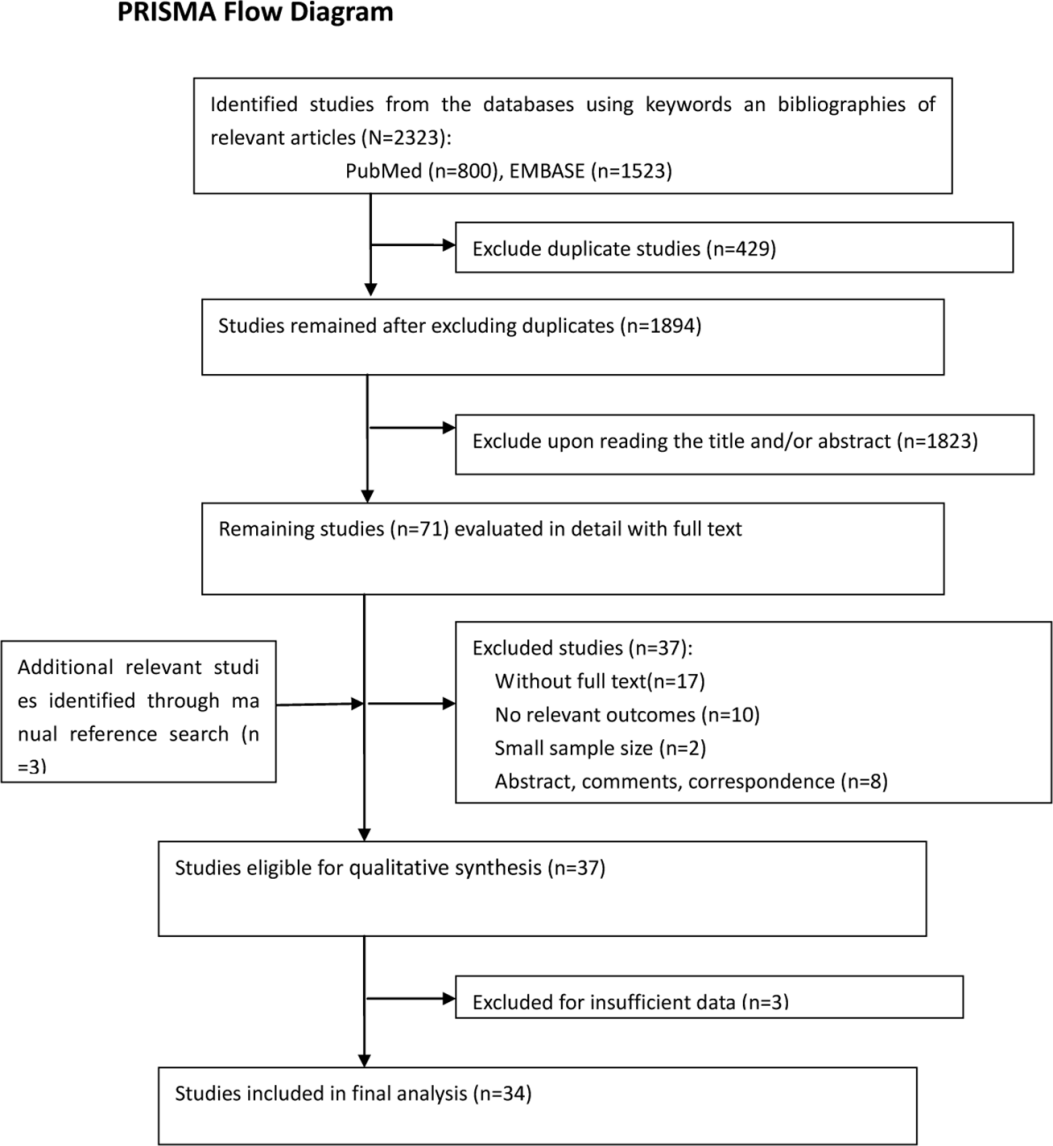
Flowchart of the study selection

The initial literature search generated 2323 citations. We included 34 studies eventually, which reported at least one of the outcomes of interest. The selection process of the studies is presented in Figure 1. Overall, the total number of patients in this meta-analysis was 29402. The median prevalence rates of BC with *BRCA1* and *BRCA2* mutations were 14.5% and 8.3%, respectively. *BRCA1* mutation was reported in 26 studies and *BRCA2* mutation was reported in 15 studies, while four studies reported the mixed mutation (*BRCA1/2* mutation). All studies were published between 1996 and 2014. The basic characteristics of the 34 included studies are shown in Table 1. The quality of the 34 included studies was generally high, as shown in Table 1 and online Supplementary Appendix S2.

**Table 1:** Basic characteristics and results of the eligible studies

First author (Year)	No. of patients	Mutant BRCA1/2 No.	Median/mean age, y	Stage	Mutation detect method	Treatment regimen	Survival end points	Median/mean follow up period (years)	Survival analysis	Adjusted varibles	Study quality
Marcus(1996) [40]	138	BRCA1 72	BRCA1 42.8; noncarriers 47.1	I-IV	NR	NR	RFS,BCSS	carriers 3.6; noncarriers 5.0	multivariate	stage	5
Foulkes(1997)[12]	112	BRCA1 12	carriers 45.2; noncarriers 52.4	I-III	PCR,sequencing	NR	DFS, BCSS	carriers 3.07; noncarriers 3.53	NR	NR	6
Johannsson(1998)[24]	152	BRCA1 40	BRCA1 43.5; noncarriers 44.9	I-III	PTT, SSCP	S/radio/chemo	OS	8	multivariate	age,stage	6
Gaffney(1998)[23]	17446	BRCA1 30; BRCA2 20	BRCA1 49.5; BRCA2 42	I-IV	full sequencing	S/radio/chemo/endoc	OS	BRCA1 9.8; BRCA2 7.5	multivariate	age,date of diagnosis,tumor size	6
Robson(1998)[25]	91	BRCA1/2 30	carriers 36; noncarriers 37	I-IV	Sequencing	S/radio/chemo/endoc	RFS	5.25	multivariate	stage,Axillary node,	6
Ansquer(1998)[22]	123	BRCA1 15	BRCA1 30; noncarriers 32	NR	NR	NR	OS	mean 3.58	NR	NR	3
Verhoog(1998)[26]	182	BRCA1 36	40	I-IV	PTT	NR	DFS,OS	NR	NR	age and year of diagnosis	5
Verhoog(1999)[27]	140	BRCA2 28	46	I-III	PTT		DFS,OS	NR	NR	age and year of diagnosis	5
Foulkes(2000)[53]	115	BRCA1 16	BRCA1 46.1; noncarriers 40	I-III	PCR,Sequencing	S/chemo	OS,BCSS	6.33	multivariate	age,tumor size,nuclear grade,estrogen receptor	8
Stoppa-Lyonnet(2000)[14]	183	BRCA1 40	BRCA1 41.1;noncarriers 42.9	I-III	DGGE	S/radio/chemo	OS	4.83	multivariate	age,menopausal status	6
Loman(2000)[28]	268	BRCA2 54	BRCA2 45.6; noncarriers 45.6	I-IV	NR	NR	OS,BCSS	BRCA2 8.1; noncarriers 8.9	multivariate	clinical stage, lymph node status and bilateral disease,	7
Hamann(2000)[13]	85	BRCA1 36	carriers 37.5; noncarriers 47	I-IV	SSCP,PTT,PCR,HA,sequencing	NR	OS,DFS	5.63	multivariate	age,bilaterality,mutation status	7
Chappuis(2000)[54]	202	BRCA 32	carriers 53.7; noncarriers 48		PCR,sequencing	s/chemo	DFS	NR	multivariate	age,tumor size,ER status,nuclear grade	6
Moller(2002)[15]	241	BRCA1 36	mean 49.0	I-III	NR	NR	OS	3.1	multivariate	grade and oestrogen receptor status	6
Goffin(2003)[16]	278	BRCA1 30	BRCA1 46.7; BRCA1/2 53.8	I-III	SSCP,PCR and DS	S/chemo	OS,DFS	8	NR	NR	7
Robson(2004)[17]	434	BRCA1 37	age 65 years or less	NR	PCR and DS	S/radio/chemo/endoc	BCSS	9.67	multivariate	Tumor size,Axillary node,Age,Chemotherapy	8
El-Tamer(2004)[29]	487	BRCA1 30; BRCA2 21	BRCA1 48.4 BRCA2 48.9	I-IV	PCR and HA	S/radio/chemo	OS,DSS	brca1 4.03; brca2 4.08	NR	NR	7
Veronesi(2005)[41]	125	BRCA1 9; BRCA2 30	BRCA-WT45.3 ; BRCA+42.3	I-IV	NR	S/chemo/endoc	OS,EFS	5.75	multivariate	age (one-year age group) and tumour grade	6
Brekelmans(2006)[18]	616	BRCA1 170	mean 41	NR	DGGE,DS,PTT,MLPA	S/radio/chemo/endoc	DFS,OS,BCSS	5.1	multivariate	tumour stage, morphology, histologic grade, estrogen receptor status, administration of systemic treatment, and B(S)O	8
Rennert(2007)[31]	1317	BRCA1 76; BRCA2 52	BRCA1 52.1; BRCA2 56.7	I-IV	NR	S/chemo	OS,BCSS	16	multivariate	age,tumor size,lymph node status,status with respect to metastasis	7
Bonadona(2007)[30]	226	BRCA1 15	under age 46	I-IV	DHPLC,HA	S/radio/chemo/endoc	BCSS,RFS	6.83	multivariate	NR	7
Moller(2007)[19]	381	BRCA1 71; BRCA2 22	BRCA1 43.9; BRCA2 46.2	I-III	NR	NR	OS	4.74	NR	NR	5
Brekelmans(2007)[55]	1019	BRCA1 170; BRCA2 90	BRCA1 42; BRCA2 44	I-III	DGGE,PTT,MLPA	s/chemo/endoc	DFS,OS,BCSS	4.3	multivariate	age,stage,treatment,oestrogen receptor status,morphology,histologic grade	7
Budroni(2009)[32]	508	BRCA2 44	median 55	I-IV	DHPLC,Sequencing	NR	OS	mean less than 5	multivariate	age	6
Lee(2011)[34]	117	BRCA1 46	BRCA1 39.3;noncarriers 51.3	I-III	HA	S/radio/chemo/endoc	BCSS, FFDM	Carriers 6.42; noncarriers 6.25	multivariate	age,stage	7
Gonzalez-Angulo(2011)[42]	77	BRCA15	carriers 45; noncarriers 53	I-III	NR	S/radio/chemo	RFS,OS	3.58	multivariate	Race,Age,Menopausal status,Histology,Pathological stage,Nuclear grade et al	6
Bayraktar(2011)[56]	227	BRCA1 94; BRCA2 20	carriers 41; noncarriers 40	I-III	NR	S/radio/chemo	RFS,OS	3.4	multivariate	disease stage,age	5
Arun(2011)[33]	269	BRCA1 55 BRCA2 21	carriers 41; noncarriers 40	I-IV	NR	S/radio/chemo/endoc	RFS,OS	3.2	multivariate	age, clinical tumor stage, ER status, nuclear	7
Goodwin(2012)[35]	1715	BRCA1 94; BRCA2 72	BRCA1 39.9; BRCA2 42.2	I-IV	DHPLC,PTT,Full sequencing	S/radio/chemo/endoc	RFS,OS	7.9	multivariate	age, tumor stage,grade, nodal status, hormone receptors, year of diagnosis	8
Bayraktar(2013)[20]	195	BRCA1 30; BRCA2 11	BRCA1 44.2	IV	NR	S/radio/chemo/endoc	OS,PFS,RFS	2.8	multivariate	N3 disease,nuclear grade 3,TN tumors, received bisphosphonates	6
Tryggvadottir(2013)[37]	1052	BRCA2 87	carriers 49.5; noncarriers 57.6	I-III	NR	NR	BCSS	9.5	multivariate	birth, year of diagnosis, size, nodal status, grade and ER status	6
Huzarski(2013)[36]	524	BRCA1 41	mean less than 45	I-III	NR	S/radio/chemo/endoc	OS	mean 7.4	multivariate	NR	6
SAMBIASI(2014)[57]	136	BRCA1 33; BRCA2 17	carriers 40.5; noncarriers 41	I-IV	DHPLC and DS	S/radio/chemo	OS,DFS	6.5	multivariate	lymph node status,tumor size and surgery	6
Nilsson(2014)[21]	221	BRCA20	carriers 34.5; noncarriers 37.0	I-III	DHPLC,SSCP,PTT	S/radio/chemo/endoc	OS	carriers 17.8; noncarriers 19.1	multivariate	age, tumor stage and chemotherapy	7

Abbreviations: PTT = protein truncation test; SSCP = single-strandson-formationalsolymorphism; DGGE = denatured gradient gel electrophoresis; HA = heteroduplex analysis; DHPLC=denaturing high performance liguld chromatography; DS = direct sequencing; MLPA = multiples ligation-dependent probe amplification; S = surgery; radio = radiotherapy; chemo = chemotherapy; endo = endocrinotherapy; OS = overall survival; BCSS= cancer-specific survival; EFS = event-free survival; FFDM= Freedom from distant metastasis ; RFS = recurrence-free survival; DFS = disease-free survival;

### Survivors for BRCA1-mutation carriers with BC

**Figure 2:**
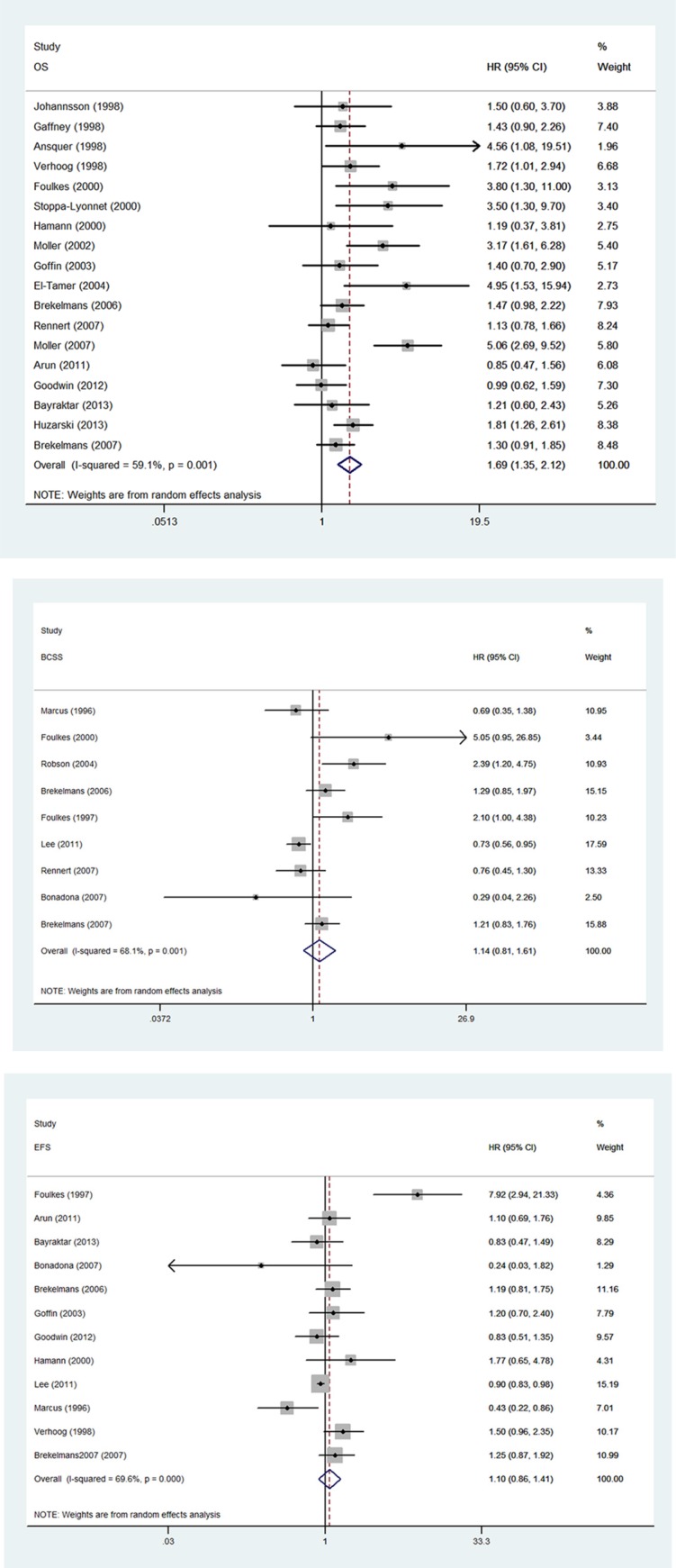
Forest plot showing the association between BRCA1 mutation and survival **(A)** Forest plot showing the association between BRCA1 and OS. **(B)** Forest plot showing the association between BRCA1 and BCSS. **(C)**. Forest plot showing the association between BRCA1 and EFS.

Among 26 studies reporting *BRCA1* mutations, 18 of these included extractable data on OS, nine on BCSS and 12 on EFS. Compared with non-carriers, BC patients with *BRCA1* mutation were significantly associated with worse OS. The pooled HR was 1.69 (95% CI 1.35 to 2.12, *p* < 0.001; *I*^2^ = 59.1%) (Figure 2A). However, we found no association between *BRCA1* mutation with a poor BCSS (HR = 1.14, 95% CI 0.81 to 1.61, *p* = 0.448; *I*^2^ = 68.1%) (Figure 2B) or EFS (HR = 1.10, 95% CI 0.86 to 1.41, *p* = 0.438; *I*^2^ = 69.6%) (Figure 2C).

The results of subgroup analysis for the association between *BRCA1* mutation and OS, BCSS, and EFS are demonstrated in Table 2. *BRCA1* was significantly associated with worse OS for studies investigating European populations (HR = 2.03, 95% CI 1.51 to 2.73, *p* < 0.001) and studies with inclusion period before 1995 (HR = 1.55, 95% CI 1.13 to 2.12, *p* = 0.007). When the analysis was stratified according to treatment with or without endocrinotherapy, the pooled HR were 1.33 (95% CI 1.11 to 1.60, *p* = 0.014) and 2.0 (95% CI 1.21 to 3.32, *p* = 0.007), respectively.

**Table 2:** Subgroup analyses of the relationships between *BRCA1* mutation and (A) OS (B)EFS or (C) BCSS

(A)					
OS Subset	HR 95% CI	*P* value	Degree of heterogeneity (*I*^2^ statistics; %)	*P*_Interaction_	No. of Studies
Total	1.69 (1.35 to 2.12)	< 0.001	59.1		18
Age of patients					
< 45	1.82 (1.33 to 2.50)	< 0.001	65.2	0.381	9
≥45	1.91 (1.10 to 3.53)	0.213	72.3		4
Sample size					
< 200	1.89 (1.32 to 2.70)	< 0.001	14.6	0.237	6
≥200	1.62 (1.23 to 2.13)	0.001	69.9		11
Years of follow-up					
< 5	1.94 (1.20 to 3.15)	0.012	66.6	0.918	7
≥ 5	1.62 (1.22 to 2.16)	0.001	61.7		10
Initial inclusion period					
Before 1995	1.55 (1.13 to 2.12)	0.007	22.4	0.088	7
After 1995	1.21 (0.83 to 1.77)	0.316	53.5		4
Country of origin					
USA	1.41 (0.98 to 2.03)	0.063	51.4	0.016	7
Europe	2.03 (1.51 to 2.73)	< 0.001	57.2		10
Asian	1.13 (0.77 to1.65)	0.526	−		1
Treatment				0.001	
Without endoc	2.0 (1.21 to 3.32)	0.007	57.6		6
With endoc	1.33 (1.11 to 1.60)	0.014	11.8		7

(B)					
EFS Subset	HR 95%CI	*P* value	Degree of heterogeneity (*I*^2^ statistics; %)	*P*_Interaction_	No. of Studies
Total	1.10 (0.86 to 1.41)	0.438	69.6		12
Age of patients					
< 45	0.98 (0.69 to 1.39)	0.892	61.6	< 0.001	6
≥45	7.92 (2.94 to 21.33)	−	0		1
Sample size					
< 200	1.22 (0.75 to 2.01)	0.422	83.1	0.105	6
≥ 200	1.10 (0.90 to 1.35)	0.347	0		6
Years of follow-up					
< 5	1.21 (0.65 to 0.26)	0.555	83.2	0.045	5
≥ 5	0.96 (0.81 to1.14)	0.661	18.7		6
Initial inclusion period					
Before 1995	1.52 (0.71 to 3.24)	0.280	83.1	0.094	5
After 1995	0.90 (0.68 to 1.20)	0.480	0		4
Country of origin		0.885	75.7		
USA	1.03 (0.72 to 1.46)	0.031	0	0.005	7
Europe	1.29 (1.02 to1.60)				5
Treatment				0.053	1
Without endoc	1.20 (0.65 to 2.22)	0.562	−		7
With endoc	0.95 (0.84 to 1.08)	0.429	11.6		

(C)					
BCSS Subset	HR 95%CI	*P* value	Degree of heterogeneity (*I*^2^ statistics; %)	*P*_Interaction_	No. of Studies
Total	1.14 (0.81 to 1.61)	0.448	68.1		9
Age of patients					
< 45	1.08 (0.78 to 1.50)	0.644	67.4	0.056	8
≥ 45	−				0
Sample size					
< 200	1.18 (0.60 to 2.32)	0.633	74.4	0.031	4
≥ 200	1.19 (0.81 to 1.74)	0.369	54.1		5
Years of follow-up					
< 5	1.19 (0.71 to 2.01)	0.508	57.5	0.177	3
≥ 5	1.13 (0.70 to 1.83)	0.622	73.1		6
Initial inclusion period					
Before 1995	1.23 (0.61 to 2.48)	0.565	68.8	0.481	4
After 1995	0.29 (0.04 to 2.18)	0.229	−		1
Country of origin					
USA	1.40 (0.73 to 2.70)	0.315	79.8	0.194	5
Europe	1.21 (0.92 to 1.60)	0.180	0.8		3
Asian	0.76 (0.45 to1.29)	0.311	−		1
Treatment					
Without endoc	1.65 (0.27 to 10.22)	0.591	77.7	0.789	2
With endoc	1.13 (0.74 to 1.75)	0.570	74.1		5

Abbreviations: OS = overall survival; EFS = event-free survival; BCSS = cancer-specific survival; endo = endocrinotherapy; HR = hazard ratio; CI = confidence interval.

As for BCSS, no significant difference between *BRCA1* carriers and non-carriers was observed. The pooled HR for patients with and without endocrinotherapy were 1.13 (95% CI 0.74 to1.75, *p* = 0.570) and 1.65 (95% CI 0.27 to10.22, *p* = 0.591), respectively. *BRCA1* was associated with a worse EFS in studies performed in European countries (HR = 1.29, 95% CI 1.02 to 1.61, *p* = 0.031). The pooled HR for patients with and without endocrinotherapy were 0.95 (95% CI 0.84 to 1.08, *p* = 0.429) and 1.20 (95% CI 0.65 to 2.22, *p* = 0.562), respectively.

### Survivors for *BRCA2*-mutation carriers with BC

**Figure 3:**
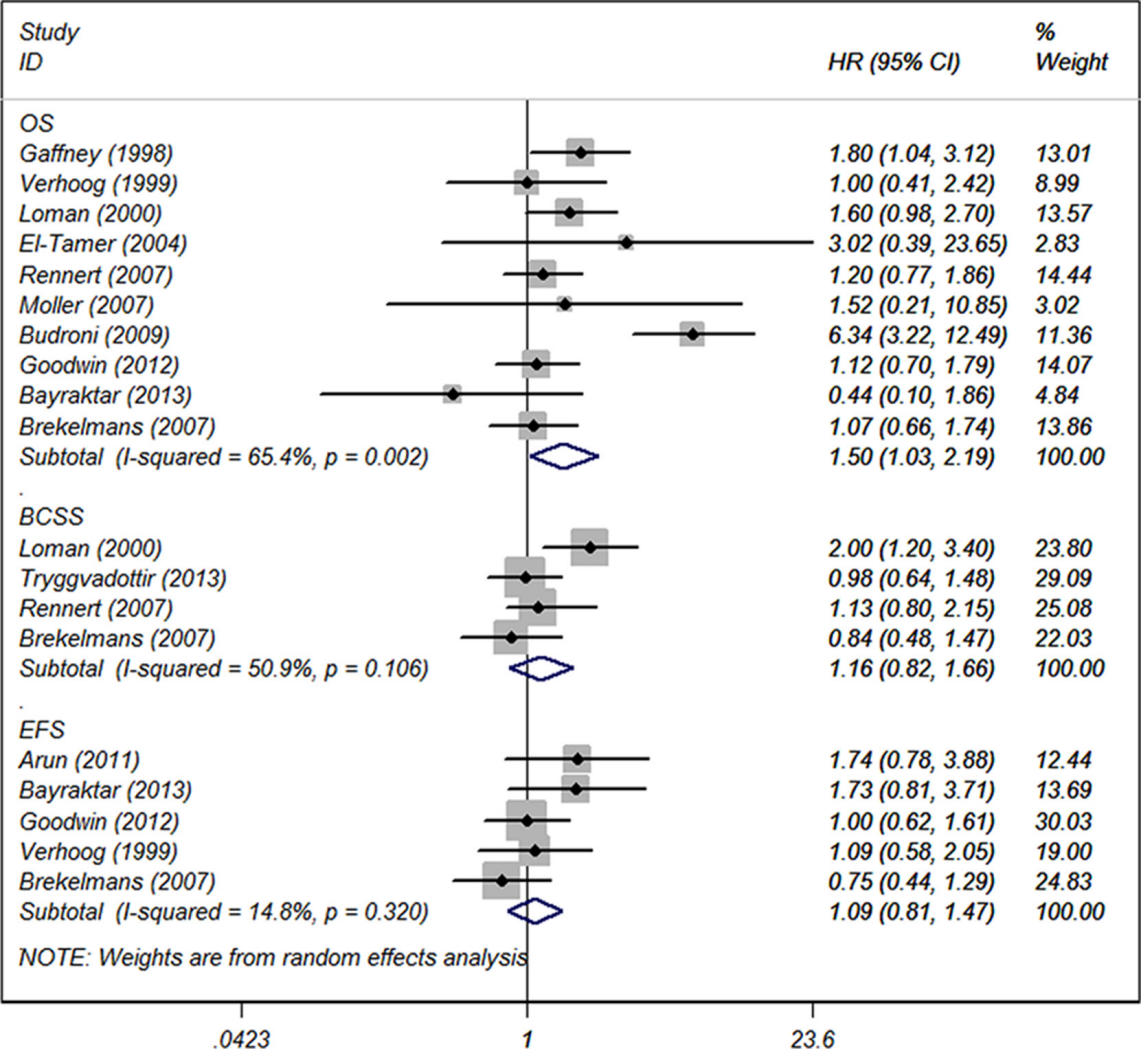
Forest plot showing the association between BRCA2 and OS, BCSS and EFS

Among 15 studies reporting *BRCA2* mutation, 10 of these reported data on OS, four on BCSS and five on EFS. Compared with non-carriers, BC patients with *BRCA2* mutation were significantly associated with worse OS. The pooled HR was 1.50 (95% CI 1.03 to 2.19, *p* = 0.034; *I*^2^ = 65.4%) (Figure 3). However, *BRCA2* mutation was not associated with poor BCSS (HR 1.16, 95% CI 0.82 to 1.66, *p* = 0.401; *I*^2^ = 50.9%) or EFS (HR 1.09, 95% CI 0.81 to 1.47, *p* = 0.558; *I*^2^ = 14.8%). The result of subgroup analysis for the association between *BRCA2* mutation and OS is demonstrated in Table 3. Significant worse OS was observed in subgroups with older age (45 years or older) (HR = 1.43, 95% CI 1.09 to 1.87, *p* = 0.009), study sample size larger than 200 (HR = 1.68, 95% CI 1.12 to 2.52, *p* = 0.012), and those with a follow up period more than 5 years (HR = 1.37, 95% CI 1.07 to 1.74, *P* = 0.012).

**Table 3:** Subgroup analyses of the relationships between *BRCA2* mutation and (A) OS (B) EFS or (C) BCSS

(A)					
OS Subset	HR 95% CI	*P* value	Degree of heterogeneity (*I*^2^ statistics; %)	*P*_Interaction_	No. of Studies
Total	1.50 (1.03 to 2.19)	0.034	65.4		9
Age of patients					
< 45	1.14 (0.72 to 1.80)	0.587	0	0.002	2
≥45	1.91 (1.10 to 3.53)	0.009	0		5
Sample size					
< 200	0.80 (0.38 to1.71)	0.568	0	0.112	2
≥ 200	1.62 (1.23 to 2.13)	0.011	70.3		7
Years of follow-up					
< 5	2.51 (0.36 to 12.81)	0.400	81.1	0.337	3
≥ 5	1.37 (1.07 to 1.74)	0.012	0		5
Initial inclusion period					
Before 1995	1.47 (1.02 to 2.11)	0.039	9.8	0.282	2
After 1995	1.91 (0.82 to 4.43)	0.133	84.8		4
Country of origin					
USA	1.30 (0.80 to 2.12)	0.296	33.2	0.346	4
Europe	2.10 (0.85 to 5.18)	0.106	78.6		4
Asian	1.20 (0.77 to 1.87)	0.418	−		1
Treatment					
Without endoc	1.25 (0.81 to 1.92)	0.311	0	0.029	2
With endoc	1.22 (0.71 to 2.11)	0.466	47.9		7

(B)					
EFS Subset	HR 95% CI	*P* value	Degree of heterogeneity (*I*^2^ statistics; %)	*P*_Interaction_	No. of Studies
Total	1.09 (0.81 to 1.47)	0.558	14.8		5
Age of patients					
< 45	0.88 (0.62 to 1.26)	0.487	0	0.130	2
≥ 45	1.09 (0.58 to 2.05)	0.789	−		1
Sample size					
< 200	1.32 (0.81 to 2.14)	0.268	0	0.333	2
≥ 200	1.01 (0.67 to 1.51)	0.969	31.6		3
Years of follow-up					
< 5	1.24 (0.68 to 2.26)	0.491	56.0	0.928	1
≥ 5	1.00 (0.62 to 1.61)	1.000	−		3
Initial inclusion period					
Before 1995	1.09 (0.58 to 2.05)	0.789	−	0.285	1
After 1995	1.28 (0.87 to 1.89)	0.203	8.7		3
Country of origin					
USA	1.28 (0.87 to 1.89)	0.203	8.7	0.189	3
Europe	0.88 (0.58 to 1.32)	0.532	0		2
Treatment				0.971	
Without endoc	−	−	−		−
With endoc	1.12 (0.76 to 1.65)	0.561	8.7		4

(C)					
BCSS Subset	HR 95% CI	*P* value	Degree of heterogeneity (*I*^2^ statistics; %)	*P*_Interaction_	No. of Studies
Total	1.16 (0.82 to 1.66)	0.401	50.9		4
Age of patients					
< 45	0.99 (0.75 to 1.30)	0.926	0	0.019	3
≥ 45	2.00 (1.19 to 3.37)	0.009	−		1
Sample size					
< 200	−	−	−	0	0
≥ 200	1.16 (0.82 to 1.66)	0.401	50.9		4
Years of follow-up					
< 5	0.84 (0.48 to 1.47)	0.541	−	0.216	1
≥ 5	1.28 (0.84 to 1.94)	0.249	56.4		3
Initial inclusion period					
Before 1995	1.04 (0.76 to 1.43)	0.809	0	0.052	2
After 1995	2.00 (1.19 to 3.37)	0.009	−		1
Country of origin					
USA	−	−	−	0.924	0
Europe	1.18 (0.71 to 1.94)	0.523	67.2		3
Asian	1.13 (0.69 to 1.85)	0.628	−		1
Treatment					
Without endoc	1.13 (0.69 to 1.85)	0.628	−	0.419	1
With endoc	0.84 (0.48 to 1.47)	0.541	−		1

Abbreviations: OS = overall survival; EFS = event-free survival; BCSS = cancer-specific survival; endo = endocrinotherapy; HR = hazard ratio; CI = confidence interval.

### Survivors for BRCA1/2-mutation carriers with BC

This group included seven studies that reported *BRCA* mutations without further specifying *BRCA1* or *BRCA2* mutation. However, *BRCA* mutations had no significant association with OS (HR = 1.21, 95% CI 0.73 to 2.00, *p* = 0.045) or EFS (HR = 0.94, 95% CI 0.60 to 1.48, *p* = 0.787).

### Sensitivity analysis and publication bias

**Figure 4:**
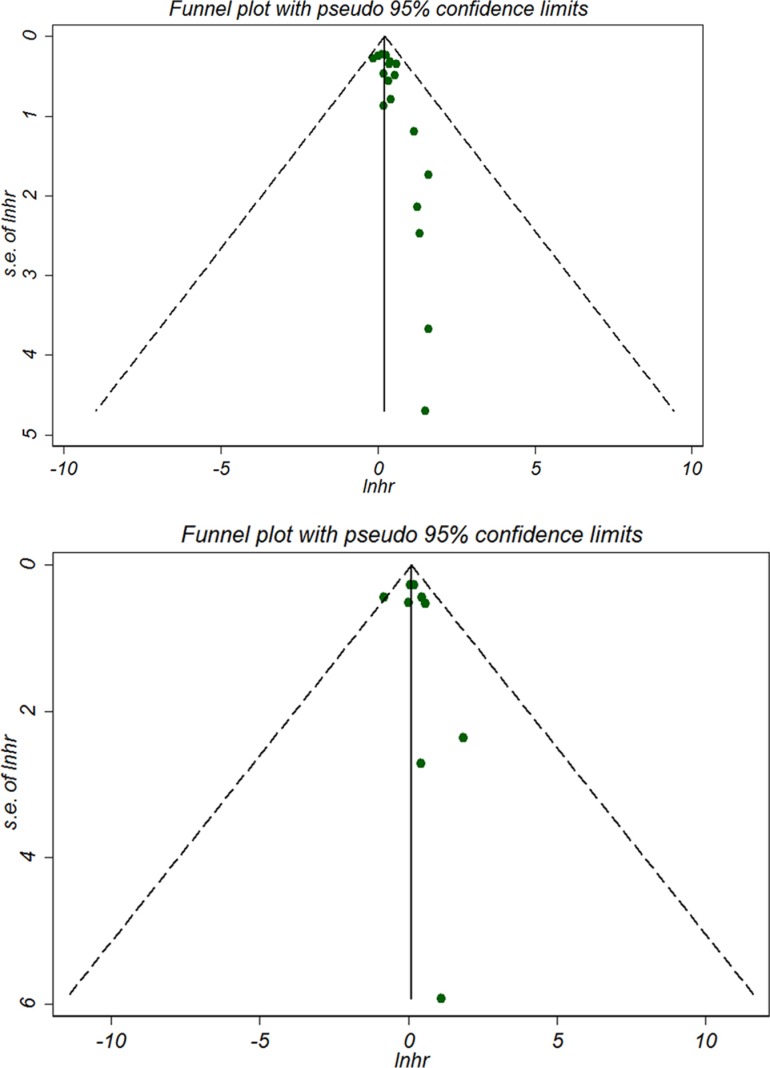
Begg's forest plot for OS of breast cancer with BRCA1 mutation (A) and BRCA2 mutation (B)

For OS in *BRCA1* mutation subset, the funnel plot suggested a possible publication bias (Figure 4A) (Begg's test *P* = 0.150 and Egger's test *P* = 0.012). Sensitivity analysis indicated that exclusion of each of the studies did not largely alter the summary estimate, which was generally consistent with the results of the subgroup analyses (Table 2A). For OS in *BRCA2* mutation subset, no evidence of publication bias was noted (Figure 4B) (Begg's test *P* = 0.474 and Egger's test *P* = 0.607). As for other survival outcomes of *BRCA* mutations, it is difficult to confirm the existence of publication bias due to the limited number of included studies. Furthermore, we also observed statistically significant association of tumor *BRCA1* and *BRCA2* mutations with OS (*BRCA1*: adjusted HR 1.50, 95% CI 1.16 to 1.93, *P* = 0.079; *BRCA2*:adjusted HR 1.50, 95% CI 1.03 to 2.19, *P* = 0.079), but not with BCSS or EFS in breast cancer patients (Supplementary Table S1) using trim and filled method to test the internal validity, which was consistent with the primary analyses.

## DISCUSSION

The mutation rate of *BRCA1* was about 1/883 in the majority of white people. However, the rate can be as high as one percent in certain populations such as the Northern European Jews [52]. *BRCA2* gene mutation is not common but can be higher in certain populations. For example, 6174ΔT specific mutation was seen in 1.5 percent of the northern European Jews, while another mutation 999 del 5 occurs in 0.6 percent of Icelanders [53]. Although our meta-analysis showed that the mutation rates of *BRCA1* and *BRCA2* were 14.5% and 8.3% respectively, the result may not represent the rates in general population as the data were originated mostly from large or small regional studies rather than global cohort.

Our meta-analysis indicated that *BRCA* mutation carriers with BC had different clinical outcome from non-carriers. Both *BRCA1* and *BRCA2* mutation are associated with reduced OS. But our study did not indicate that BC patients with *BRCA1* and *BRCA2* mutations had improved BCSS or EFS compared to those without *BRCA1* or *BRCA2* mutations.

Our subgroup analysis demonstrated that patients with endocrinotherapy had improved OS compared to those without endocrinotherapy (*P*_interation_ = 0.001) in *BRCA1* carriers. It is partly due to the fact that BCs with *BRCA1* mutations are more sensitive to endocrinotherapy, though it is reported that most of the *BRCA1*-related BCs are estrogen receptor negative and adjuvant endocrinotherapy is usually ineffective in the absence of estrogen receptors.

Though lack data on endocrinotherapy for BCs, several studies have reported special patterns that *BRCA* mutation-associated BCs are sensitive to some specific chemotherapies [57–61], especially sensitive to those drugs inhibiting poly (ADP-Ribose) polymerase (PARP) [62]. Based on these findings, it is promising that *BRCA*mutation status could guide future chemotherapy in BCs. It was also reported that ovarian cancer could be more sensitive to platinum based chemotherapy than non-carriers [63]. Further trials could be conducted to test endocrinotherapy on the prognostic effects in BCs.

The studies performed in European populations had statistically worse OS and EFS compared with studies performed in non-European populations. This may be due to the higher *BRCA1* mutation rate in European population. The studies with the inclusion period after 1995 showed a slight improvement in OS, BCSS and EFS in *BRCA1* carriers, but only statistically significant for EFS. This is perhaps the result of the development of medical standard (for example, the improvement of the treatment standard). Subgroup analysis among *BRCA2*-mutation carriers found that older age (≥ 45 years) was associated with statistically worse OS, compared with younger age. Studies with larger sample size (greater than 200), longer follow-up duration (longer than 5 years) were also associated with worse OS, but none of these had statistical significance.

The effect of *BRCA1* mutation on outcomes of BC patients may differ from *BRCA2* mutation as a result of different molecular mechanisms of tumorgenesis. Although the specific molecular mechanisms are unclear currently, several studies have shown different clinical behaviors of *BRCA1* and *BRCA2* carriers. For example, patients with *BRCA1*-related BC were usually younger, less than 40 years old typically. Our meta-analysis shows that the average age of *BRCA1*-mutation carriers was 43 years old. These patients often develop invasive cancer directly without precancerous stage (such as ductal carcinoma *in situ*). Immunohistochemically, BCs with *BRCA1*mutation often stain positive for CK5/6, negative for ER, PR and HER-2, and often overexpress P53. For *BRCA2*-related BCs, the histologic grade is often higher than that of in sporadic BCs. But the expression of ER/PR was similar with non-mutation BCs and there is no increase in expression of P53.

Compared with the previous meta-analyses [54, 56], ours has several strengths, including the broad search strategy with comprehensive search terms in major databases, the largest sample size of over 297000 patients (having a much higher level of statistical power) and sufficient subgroup analyses. Thus, this updated meta-analysis can reasonably systematically quantify the association between *BRCA*-mutations and BC outcomes. Furthermore, all the data were stratified according to OS, EFS, and BCSS, and were analyzed independently, which was more comprehensive than previous ones with only two outcome measures (OS and PFS). By evaluating the effect of *BRCA1* and *BRCA2* mutation on prognosis, our study supports the hypothesis that both *BRCA1* and *BRCA2*-mutation carriers has worse OS and could be independent prognostic factors for BC. What's more, one limitation of the previous meta-analyses lies in that they have not thoroughly investigated the influence of publication bias. In our study, we used Begg's test, Egger's test and sensitivity analysis to test the influence of publication bias and confirmed the robutness of the results. However, as evidence accumulated, such findings should be interpreted with caution.

As with any meta-analysis, several limitations of our study should be addressed. First, the characteristics of the included population varied among studies (sample size, patient age, disease stage and duration of follow-up), which to some extent were contributory factors to the heterogeneity. Second, the measurement methods of*BRCA*-mutations were different among studies, which may result in substantial heterogeneity. Third, the analysis was based on published studies without including grey literature, which might have limitations in publication or selection bias. In addition, for the variation among different cancer stage and prognosis and multiple treatment strategies applied rather than a standardized one, and most studies used multivariate Cox proportion hazard models with different adjusted variables to deal with the estimates, a certain degree of heterogeneity do exist in this study.

One previous meta-analysis has assessed the association between *BRCA*-mutation and survival among patients with BC based on 11 observational studies [54] and didn't find a statistically significant relationship between *BRCA2*-mutation and OS. However, it reported a worse short-term progress-free survival in *BRCA1*-mutation carriers. However, through a more comprehensive and thorough literature search and this study has yielded a total of 34 studies, our analysis found both *BRCA1* mutations and *BRCA2* mutations were associated with worse OS. However, we didn't find significant association between *BRCA1* mutation and EFS. Furthermore, compared with the last published meta-analysis [56], we have added 19 new studies. We involved a total of 297,402 patients with BC from 34 studies, compared with 10,016 patients from 13 studies, which was a much larger sample size and added greater statistical power to the analysis.

Our study indicated that *BRCA* mutations were associated with poor OS but did not translate into poor BCSS. It is due to some unmeasured confoundings given the observational nature of the included studies which we cannot fully account for bias. First, only nine studies investigated the association between *BRCA* mutations and BCCS with limited number of sample size (Table 1). Therefore, statistical significance may not be reached due to limited statistical power. Further large-scale studies should be warranted to verify the results of the analyses. Second, the adjusted variables for OS and BCSS varied among the included studies (Table 1), which was an inherent limitation in this study-level meta-analysis, combining survival estimates from study-level results as opposed to individual patient results. Since the study-level meta-analysis cannot fully investigate the interaction among different individual prognostic factors. Compared with an individual patient data approach, the effect estimates provided for *BRCA* mutations in this meta-analysis cannot be fully adjusted for other potential influential factors, such as tumor stage, grade, nodal status, hormone receptors or systemic treatment. The survival estimates for OS and BCSS were abstracted from separate analyses with different statistical approaches, instead of being obtained from the same statistical model based on patient level data. Thus, meta-analyses of individual patient data with similar adjusted variables for both OS and BCSS are strongly advocated in the future.

The results of further subgroup analyses showed that the inter-study heterogeneity decreased substantially for most of the investigated variables, which indicated that the heterogeneity could be explained partly by those investigated factors (Table 2 and Table 3). However, in some cases, heterogeneity remained considerable with I^2^ more than 50%. It has been reported that nearly 25% of all meta-analyses having I^2^ more than 50%, which is a common challenge of systematic reviews [55].

Although the *BRCA1/2* mutations or other investigated factors identified give informative survival association on BC patients, causality cannot be inferred due to the nature of observational study. Besides, the estimates abstracted from the original reports are from the combined effects of both univariate and multivariate analysis. Therefore, we cannot draw definite conclusions due to such heterogeneity because the interaction among the investigated factors cannot be fully determined.

Based on the results of this comprehensive meta-analysis, *BRCA1* and *BRCA2* mutations are associated with worse OS in women with BC. An improved survival was observed in BC patients who had *BRCA1* mutation and treated with endocrinotherapy. The results may have therapeutic and prognostic implications important for BRAC mutation carriers with breast cancer. Further studies should be focused on the association between BC survival and *BRCA* mutations stratified by ER/PR status.

## MATERIALS AND METHODS

### Literature search and study selection

We searched the PubMed and EMBASE databases for studies published up to March 2015. Detailed search terms and strategies for the two databases are provided in Supplementary Appendix S1. In addition, we reviewed the references of eligible articles to identify any relevant publications that were not identified during the preliminary literature searches.

The studies were included in the current study if they met the following criteria: (1) being an original study for women with breast cancer; (2) investigating the prognostic outcomes of *BRCA* mutation carriers versus non-carriers; and (3) providing hazard ratios (HR) with 95% confidence interval (CI) or related data for calculating them. The studies with only abstracts or unpublished data were excluded from the analysis. If multiple publications were identified from the same population, the publication with the most informative information or the largest sample size was included.

### Data extraction and quality assessment

The data extraction was conducted by two authors independently and cross checked to make sure for accuracy. Any uncertainty about the extracted data was deliberated and resolved by agreement between the authors. OS was used as the primary outcome measure which was defined as the time from initial breast cancer diagnosis to death due to any causes. Breast cancer-specific survival (BCSS) and event-free survival (EFS) were set as the secondary outcome measures. Breast cancer-specific survival (BCSS) was defined as the time from initial breast cancer diagnosis to death due to breast cancer. Both distant disease-free survival (DFS) and recurrence-free survival (RFS) were defined as the interval between surgical resection of the primary breast cancer and the first recurrence of the tumor. Freedom from distant metastasis (FFDM) was defined from the date of initial breast cancer diagnosis until the date of first distant metastasis. EFS was defined as the time from initial breast cancer diagnosis until the date of last follow-up evaluation, development of metachronous contralateral breast cancer, relapse of cancer, or distant metastasis, whichever occurred first. DFS, RFS and FFDM were analyzed together as EFS.

The information extracted from each study includes the first author, year of publication, country where the study was performed, duration of follow-up, number of cancer and control cases, tumor stage, adjustment variables, and hazard ratios (HRs) and 95% CI for corresponding survival outcomes. In some studies with incomplete data in publications, the authors were contacted for unreported data whenever it was feasible. HRs and corresponding 95% CIs were extracted preferentially from multivariate analyses or univariate analyses when available. Otherwise, they were calculated using the methods provided by Parmar and Tierney [45, 46].

According to the Newcastle-ottawa Scale (NOS) [47], two evaluators independently assessed and scored the methodological quality of included studies based on three aspects, that is, study design (including the selection of study population), data comparability and outcome assessment. On a scale from zero to nine, studies scored five or greater were considered to be of high quality, while those scored below five were classified as low quality.

### Statistical analysis

We used random-effects models to estimate the summary HRs for the associations between *BRCA* mutations and outcomes among BC survivors. I^2^ statistic was used to evaluate the statistical heterogeneity among studies [48]. An *I*^2^ value > 50% indicated substantial heterogeneity. The sources of potential heterogeneity among studies were explored using subgroup analysis [49]. We further analyzed the association between *BRCA1*-mutation and outcomes among subgroups of BC survivors stratified by age, residency country, sample size, treatment and follower-up period. Sensitivity analysis using trim and filled method was also applied to test the internal validity. The risk of publication bias was assessed by visually inspecting the funnel plot asymmetry as well as by using Egger's regression test [50] and Begg's rank correlation test [51]. Stata statistical software (version 12.0; Stata Corporation, College Station, TX, USA) was used to perform the meta-analysis. The *p value*s were two sided with a significance level of less than 0.05.

## SUPPLEMENTARY MATERIALS



## Abbreviations

BC: breast cancer
OS: overall survival
BCSS: cancer-specific survival
EFS: event-free survival
DFS: disease-free survival
HRs: Hazard ratios
CIs: confidence intervals
ER: estrogen receptor
PR: progesterone receptor
HER-2: human epidermal growth factor receptor-2
CK5/6: cytokeratins 5/6
FFDM: Freedom from distant metastasis
RFS: recurrence-free survival
NOS: Newcastle-ottawa Scale.

## References

[1] Hall JM, Lee MK, Newman B, Morrow JE, Anderson LA, Huey B, King M-C (1990). Linkage of early-onset familial breast cancer to chromosome 17q21. Science.

[2] Lenoir G, Lynch H, Watson P, Conway T, Lynch J, Narod S, Feunteun J (1991). Familial breast-ovarian cancer locus on chromosome 17q12-q23. The Lancet.

[3] Hall J, Friedman L, Guenther C, Lee M, Weber J, Black D, King M (1992). Closing in on a breast cancer gene on chromosome 17q. American journal of human genetics.

[4] Wooster R, Neuhausen SL, Mangion J, Quirk Y, Ford D, Collins N, Nguyen K, Seal S, Tran T, Averill D (1994). Localization of a breast cancer susceptibility gene, *BRCA2*, to chromosome 13q12–13. Science.

[5] Wang F, Fang Q, Ge Z, Yu N, Xu S, Fan X (2012). Common *BRCA1* and *BRCA2* mutations in breast cancer families: a meta-analysis from systematic review. Molecular biology reports.

[6] Antoniou A, Pharoah P, Narod S, Risch HA, Eyfjord JE, Hopper J, Loman N, Olsson H, Johannsson O, Borg Å (2003). Average risks of breast and ovarian cancer associated with *BRCA1* or *BRCA2* mutations detected in case series unselected for family history: a combined analysis of 22 studies. The American Journal of Human Genetics.

[7] King M-C, Marks JH, Mandell JB, Group NYBCS (2003). Breast and ovarian cancer risks due to inherited mutations in *BRCA1* and *BRCA2*. Science.

[8] Chen S, Parmigiani G (2007). Meta-analysis of *BRCA1* and *BRCA2* penetrance. Journal of Clinical Oncology.

[9] Bordeleau L, Panchal S, Goodwin P (2010). Prognosis of *BRCA*-associated breast cancer: a summary of evidence. Breast cancer research and treatment.

[10] Lakhani SR, Gusterson BA, Jacquemier J, Sloane JP, Anderson TJ, van de Vijver MJ, Venter D, Freeman A, Antoniou A, McGuffog L (2000). The pathology of familial breast cancer: histological features of cancers in families not attributable to mutations in *BRCA1* or *BRCA2*. Clinical Cancer Research.

[11] Narod SA, Foulkes WD (2004). *BRCA1* and *BRCA2*: 1994 and beyond. Nature Reviews Cancer.

[12] Foulkes WD, Wong N, Brunet J-S, Begin LR, Zhang JC, Martinez JJ, Rozen F, Tonin PN, Narod SA, Karp SE (1997). Germ-line BRCA1 mutation is an adverse prognostic factor in Ashkenazi Jewish women with breast cancer. Clinical Cancer Research.

[13] Lakhani SR, Gusterson BA, Jacquemier J, Sloane JP, Anderson TJ, van de Vijver MJ, Venter D, Freeman A, Antoniou A, McGuffog L (2000). The pathology of familial breast cancer: histological features of cancers in families not attributable to mutations in BRCA1 or BRCA2. Clinical Cancer Research.

[14] Hamann U, Sinn H-P (2000). Survival and tumor characteristics of German hereditary breast cancer patients. Breast cancer research and treatment.

[15] Stoppa-Lyonnet D, Ansquer Y, Dreyfus H, Gautier C, Gauthier-Villars M, Bourstyn E, Clough KB, Magdelénat H, Pouillart P, Vincent-Salomon A (2000). Familial invasive breast cancers: worse outcome related to BRCA1 mutations. Journal of Clinical Oncology.

[16] Møller P, Borg Å, Evans DG, Haites N, Reis MM, Vasen H, Anderson E, Steel CM, Apold J, Goudie D (2002). Survival in prospectively ascertained familial breast cancer: analysis of a series stratified by tumour characteristics, BRCA mutations and oophorectomy. International journal of cancer.

[17] Goffin JR, Chappuis PO, Bégin LR, Wong N, Brunet JS, Hamel N, Paradis AJ, Boyd J, Foulkes WD (2003). Impact of germline BRCA1 mutations and overexpression of p53 on prognosis and response to treatment following breast carcinoma. Cancer.

[18] Robson ME, Chappuis PO, Satagopan J, Wong N, Boyd J, Goffin JR, Hudis C, Roberge D, Norton L, Bégin LR (2004). A combined analysis of outcome following breast cancer: differences in survival based on BRCA1/BRCA2 mutation status and administration of adjuvant treatment. Breast Cancer Res.

[19] Brekelmans C, Seynaeve C, Menke-Pluymers M, Brüggenwirth H, Tilanus-Linthorst M, Bartels C, Kriege M, van Geel A, Crepin C, Blom J (2006). Survival and prognostic factors in BRCA1-associated breast cancer. Annals of oncology.

[20] Moller P, Evans DG, Reis MM, Gregory H, Anderson E, Maehle L, Lalloo F, Howell A, Apold J, Clark N (2007). Surveillance for familial breast cancer: Differences in outcome according to BRCA mutation status. International journal of cancer.

[21] Bayraktar S, Gutierrez-Barrera A, Lin H, Elsayegh N, Tasbas T, Litton J, Ibrahim N, Morrow P, Green M, Valero V (2013). Outcome of metastatic breast cancer in selected women with or without deleterious BRCA mutations. Clinical & experimental metastasis.

[22] Nilsson MP, Hartman L, Idvall I, Kristoffersson U, Johannsson OT, Loman N (2014). Long-term prognosis of early-onset breast cancer in a population-based cohort with a known BRCA1/2 mutation status. Breast cancer research and treatment.

[23] Ansquer Y, Gautier C, Fourquet A, Asselain B, Stoppa-Lyonnet D (1998). Survival in early-onset BRCA1 breast-cancer patients. The Lancet.

[24] Gaffney DK, Brohet RM, Lewis CM, Holden JA, Buys SS, Neuhausen SL, Steele L, Avizonis V, Stewart JR, Cannon-Albright LA (1998). Response to radiation therapy and prognosis in breast cancer patients with BRCA1 and BRCA2 mutations. Radiotherapy and oncology.

[25] Johannsson OT, Ranstam J, Borg A, Olsson H (1998). Survival of BRCA1 breast and ovarian cancer patients: a population-based study from southern Sweden. Journal of Clinical Oncology.

[26] Robson M, Gilewski T, Haas B, Levin D, Borgen P, Rajan P, Hirschaut Y, Pressman P, Rosen PP, Lesser ML (1998). BRCA-associated breast cancer in young women. Journal of Clinical Oncology.

[27] Verhoog L, Brekelmans C, Seynaeve C, Van den Bosch L, Dahmen G, Van Geel A, Tilanus-Linthorst M, Bartels C, Wagner A, van den Ouweland A (1998). Survival and tumour characteristics of breast-cancer patients with germline mutations of BRCA1. The Lancet.

[28] Verhoog L, Brekelmans C, Seynaeve C, Dahmen G, Van Geel A, Bartels C, Tilanus-Linthorst M, Wagner A, Devilee P, Halley D (1999). Survival in hereditary breast cancer associated with germline mutations of BRCA2. Journal of Clinical Oncology.

[29] Loman N, Johannsson O, Bendahl P-O, Dahl N, Einbeigi Z, Gerdes A-M, Borg Å, Olsson H (2000). Prognosis and clinical presentation of BRCA2-associated breast cancer. European journal of cancer.

[30] El-Tamer M, Russo D, Troxel A, Bernardino LP, Mazziotta R, Estabrook A, Ditkoff B-A, Schnabel F, Mansukhani M (2004). Survival and recurrence after breast cancer in BRCA1/2 mutation carriers. Annals of surgical oncology.

[31] Bonadona V, Dussart-Moser S, Voirin N, Sinilnikova OM, Mignotte H, Mathevet P, Brémond A, Treilleux I, Martin A, Romestaing P (2007). Prognosis of early-onset breast cancer based on BRCA1/2 mutation status in a French population-based cohort and review. Breast cancer research and treatment.

[32] Rennert G, Bisland-Naggan S, Barnett-Griness O, Bar-Joseph N, Zhang S, Rennert HS, Narod SA (2007). Clinical outcomes of breast cancer in carriers of BRCA1 and BRCA2 mutations. New England Journal of Medicine.

[33] Budroni M, Cesaraccio R, Coviello V, Sechi O, Pirino D, Cossu A, Tanda F, Pisano M, Palomba G, Palmieri G (2009). Role of BRCA2 mutation status on overall survival among breast cancer patients from Sardinia. BMC cancer.

[34] Arun B, Bayraktar S, Liu DD, Barrera AMG, Atchley D, Pusztai L, Litton JK, Valero V, Meric-Bernstam F, Hortobagyi GN (2011). Response to neoadjuvant systemic therapy for breast cancer in BRCA mutation carriers and noncarriers: a single-institution experience. Journal of Clinical Oncology.

[35] Baranyak Z, Madaras L, Tokes A, Szasz A, Szekely B, Kulka J (2011). 86 Cases of Early-onset Breast Cancer in Hungary-Retrospective Analysis of Immunohistochemistry (IHC) and Family-history Data-Assessing the Risk of Carrying BRCA1 and BRCA2 Mutation. Eur J Cancer.

[36] Lee LJ, Alexander B, Schnitt SJ, Comander A, Gallagher B, Garber JE, Tung N (2011). Clinical outcome of triple negative breast cancer in BRCA1 mutation carriers and noncarriers. Cancer.

[37] Goodwin PJ, Phillips K-A, West DW, Ennis M, Hopper JL, John EM, O'Malley FP, Milne RL, Andrulis IL, Friedlander ML (2011). Breast cancer prognosis in BRCA1 and BRCA2 mutation carriers: an International Prospective Breast Cancer Family Registry population-based cohort study. Journal of Clinical Oncology.

[38] Huzarski T, Byrski T, Gronwald J, Górski B, Domagała P, Cybulski C, Oszurek O, Szwiec M, Gugała K, Stawicka M (2013). Ten-year survival in patients with BRCA1-negative and BRCA1-positive breast cancer. Journal of Clinical Oncology.

[39] Tryggvadottir L, Olafsdottir EJ, Olafsdottir GH, Sigurdsson H, Johannsson OT, Bjorgvinsson E, Alexiusdottir K, Stefansson OA, Agnarsson BA, Narod SA (2013). Tumour diploidy and survival in breast cancer patients with BRCA2 mutations. Breast cancer research and treatment.

[40] Sambiasi D, Lambo R, Pilato B, Tommasi S, Trojano G, Kardhashi A, Digennaro M, Trojano V, Simone G, Paradiso A (2014). BRCA1/2 and clinical outcome in a monoinstitutional cohort of women with hereditary breast cancer. Oncology reports.

[41] Robson M, Levin D, Federici M, Satagopan J, Bogolminy F, Heerdt A, Borgen P, McCormick B, Hudis C, Norton L (1999). Breast conservation therapy for invasive breast cancer in Ashkenazi women with BRCA gene founder mutations. Journal of the National Cancer Institute.

[42] Marcus JN, Watson P, Page DL, Narod SA, Lenoir GM, Tonin P, Linder, Stephenson L, Salerno G, Conway TA, Lynch HT (1996). Hereditary breast cancer. pathobiology, prognosis, and BRCA1 and BRCA2 gene linkage. Cancer.

[43] Veronesi A, de Giacomi C, Magri MD, Lombardi D, Zanetti M, Scuderi C, Dolcetti R, Viel A, Crivellari D, Bidoli E (2005). Familial breast cancer: characteristics and outcome of BRCA 1–2 positive and negative cases. BMC cancer.

[44] Gonzalez-Angulo AM, Timms KM, Liu S, Chen H, Litton JK, Potter J, Lanchbury JS, Stemke-Hale K, Hennessy BT, Arun BK (2011). Incidence and outcome of BRCA mutations in unselected patients with triple receptor-negative breast cancer. Clinical Cancer Research.

[45] Parmar MK, Torri V, Stewart L (1998). Extracting summary statistics to perform meta-analyses of the published literature for survival endpoints. Statistics in medicine.

[46] Tierney JF, Stewart LA, Ghersi D, Burdett S, Sydes MR (2007). Practical methods for incorporating summary time-to-event data into meta-analysis. Trials.

[47] Stang A (2010). Critical evaluation of the Newcastle-Ottawa scale for the assessment of the quality of nonrandomized studies in meta-analyses. European journal of epidemiology.

[48] Higgins J, Thompson SG (2002). Quantifying heterogeneity in a meta-analysis. Statistics in medicine.

[49] DerSimonian R, Laird N (1986). Meta-analysis in clinical trials. Controlled clinical trials.

[50] Egger M, Smith GD, Schneider M, Minder C (1997). Bias in meta-analysis detected by a simple, graphical test. Bmj.

[51] Begg CB, Mazumdar M (1994). Operating characteristics of a rank correlation test for publication bias. Biometrics.

[52] Warner E, Foulkes W, Goodwin P, Meschino W, Blondal J, Paterson C, Ozcelik H, Goss P, Allingham-Hawkins D, Hamel N (1999). Prevalence and penetrance of BRCA1 and BRCA2 gene mutations in unselected Ashkenazi Jewish women with breast cancer. Journal of the National Cancer Institute.

[53] Thorlacius S, Struewing JP, Hartage P, Olafsdottir GH, Sigvaldason H, Tryggvadottir L, Wacholder S, Tulinius H, Eyfjörd JE (1998). Population-based study of risk of breast cancer in carriers of BRCA2 mutation. The Lancet.

[54] Lee E-H, Park SK, Park B, Kim S-W, Lee MH, Ahn SH, Son BH, Yoo K-Y, Kang D, Group KR (2010). Effect of BRCA1/2 mutation on short-term and long-term breast cancer survival: a systematic review and meta-analysis. Breast cancer research and treatment.

[55] Higgins JP, Thompson SG, Deeks JJ, Altman DG (2003). Measuring inconsistency in meta-analyses. Bmj.

[56] Zhong Q, Peng H-L, Zhao X, Zhang L, Hwang W-T (2015). Effects of BRCA1-and BRCA2-related mutations on ovarian and breast cancer survival. a meta-analysis. Clinical Cancer Research.

[57] Byrski T, Huzarski T, Dent R, Gronwald J, Zuziak D, Cybulski C, Kladny J, Gorski B, Lubinski J, Narod SA (2009). Response to neoadjuvant therapy with cisplatin in BRCA1-positive breast cancer patients. Breast Cancer Res Treat.

[58] Byrski T, Gronwald J, Huzarski T, Grzybowska E, Budryk M, Stawicka M, Mierzwa T, Szwiec M, Wisniowski R, Siolek M, Dent R, Lubinski J, Narod S (2010). Pathologic complete response rates in young women with BRCA1-positive breast cancers after neoadjuvant chemotherapy. J Clin Oncol.

[59] Byrski T, Gronwald J, Huzarski T, Grzybowska E, Budryk M, Stawicka M, Mierzwa T, Szwiec M, Wisniowski R, Siolek M, Dent R, Lubinski J, Narod S (2010). Pathologic complete response rates in young women with BRCA1-positive breast cancers after neoadjuvant chemotherapy. J Clin Oncol.

[60] Wen J, Li R, Lu Y, Shupnik MA (2009). Decreased BRCA1 confers tamoxifen resistance in breast cancer cells by altering estrogen receptor-coregulator interactions. Oncogene.

[61] Fong PC, Boss DS, Yap TA, Tutt A, Wu P, Mergui-Roelvink M, Mortimer P, Swaisland H, Lau A, O'Connor MJ, Ashworth A, Carmichael J, Kaye SB, Schellens JH, de Bono JS (2009). Inhibition of poly (ADP-ribose) polymerase in tumors from BRCA mutation carriers. N Engl J Med.

[62] Fong PC, Boss DS, Yap TA, Tutt A, Wu P, Mergui-Roelvink M, Mortimer P, Swaisland H, Lau A, O'Connor MJ, Ashworth A, Carmichael J, Kaye SB, Schellens JH, de Bono JS (2009). Inhibition of poly (ADP-ribose) polymerase in tumors from BRCA mutation carriers. N Engl J Med.

[63] Cass I, Baldwin RL, Varkey T, Moslehi R, Narod SA, Karlan BY (2003). Improved survival in women with BRCA-associated ovarian carcinoma. Cancer.

