# Supplementary material for: BRCA mutations and survival in breast cancer: an updated systematic review and meta-analysis
Source: Oncotarget. 2016 Sep 21;7(43):70113–27. doi: 10.18632/oncotarget.12158 (PMC5342539; doi:10.18632/oncotarget.12158)
Supplement: Supplementary file 1 [file oncotarget-07-70113-s001.pdf]

BRCA mutations and survival in breast cancer: an updated systematic review and meta-analysis

Supplementary Materials

Supplementary Appendix S1: Search strategy in Pubmed up till March 2015 with Daily Update (similar search run in EMBASE)

1. "Breast Neoplasms"[Mesh]
2. breast cancer*[Title/Abstract]
3. breast tumo*[Title/Abstract]
4. tumo* AND, breast[Title/Abstract]
5. breast carcinoma*[Title/Abstract]
6. neoplas* AND , breast[Title/Abstract]
7. cancer of the breast [Title/Abstract]
8. breast neoplasms [Title/Abstract]
9. cancer* AND, breast[Title/Abstract]
10. 1 OR 2 OR 3 OR 4 OR 5 OR 6 OR 7 OR 8 OR 9
11. "Mutation"[Mesh]
12. mutation*[Title/Abstract]
13. shop*[Title/Abstract]
14. saltation[Title/Abstract]
15. 11 OR 12 OR 13 OR 14
16. "Genes, BRCA1"[Mesh]
17. "BRCA1 Protein"[Mesh]
18. brca1 gene*[Title/Abstract]
19. gene* AND, brca1*[Title/Abstract]
20. brca1* protein[Title/Abstract]
21. "Genes, BRCA2"[Mesh]
22. "BRCA2 Protein"[Mesh]
23. brca2 gene*[Title/Abstract]
24. gene* AND, brca2*[Title/Abstract]
25. brca1* protein[Title/Abstract]
26. 16 OR 17 OR 18 OR 19 OR 20 OR 21 OR 22 OR 23 OR 24 OR 25
27. 10 AND 15 AND 26
28. "Survival"[Mesh]
29. "Mortality"[Mesh]
30. outcome*[Title/Abstract]
31. Survival*[Title/Abstract]
32. mortalit*[Title/Abstract]
33. prognosis[Title/Abstract]
34. 28 OR 29 OR 30 OR 31 OR 32 OR 33
35. 27 AND 34

Supplementary Appendix S2: Quality assessment of the included studies

Newcastle-Ottawa Scale for assessing the quality of studies in meta-analysis

	Selection				Comparability	Outcome			
**Study**	**Represent-ativeness of the exposed cohort**	**Selection of the non exposed cohort**	**Ascertain-ment of exposure**	**Demonstration that outcome of interest was not present at start of study**	**Comparability of cohorts on the basis of the design or analysis**	**Assess-ment of outcome**	**Was follow-up long enough for outcomes to occur**	**Adequacy of follow up of cohorts**	**Qua-lity score**
Marcus et al, 1996		★		★	★	★	★		5
Foulkes et al, 1997		★	★	★	★	★	★	★	7
Johannsson et al, 1998		★	★	★	★	★	★		6
Gaffney et al, 1998		★	★	★	★	★	★	★	7
Robson et al, 1998		★	★	★	★★	★	★		7
Ansquer et al, 1998		★	★	★	★★	★	★	★	8
Verhoog et al, 1998		★	★	★	★★	★	★	★	8
Verhoog et al, 1999		★	★	★	★★	★	★		7
Foulkes et al, 2000		★	★	★	★★	★	★		7
Stoppa-Lyonnet et al, 2000		★	★	★	★★	★	★		5
Loman et al, 2000		★	★	★	★★	★	★		7
Hamann et al, 2000		★	★	★	★	★	★	★	7
Chappuis et al, 2000		★	★	★	★★	★			6
Moller et al, 2002		★	★	★	★	★	★		6
Goffin et al, 2003		★	★	★	★	★	★	★	7
Robson et al, 2004		★	★	★	★★	★	★	★	8
El-Tamer et al, 2004		★	★	★	★	★	★	★	7
Veronesi et al, 2005		★	★	★	★★	★	★	★	6
Brekelmans et al, 2006		★	★	★	★★	★	★	★	8
Rennert et al, 2007		★	★		★★	★	★	★	7
Bonadona et al, 2007		★	★	★	★	★	★	★	7
Moller et al, 2007		★	★	★		★	★		5
Brekelmans et al, 2007		★	★	★	★★	★	★		7
Budroni et al, 2007		★	★	★	★	★	★		6
Lee et al, 2011		★	★	★	★	★	★	★	7
Gonzalez-Angulo et al, 2011		★		★	★★	★	★		6
Bayraktar et al, 2011		★		★	★	★	★		5
Arun et al, 2011		★		★	★★	★	★	★	7
Goodwin et al, 2012		★	★	★	★★	★	★	★	8
Bayraktar et al, 2013		★		★	★★	★	★		6
Tryggvadottir et al, 2013		★		★	★★	★	★		6
Huzarski et al, 2013		★		★	★	★	★	★	6
SAMBIASI et al, 2014		★	★	★	★	★	★		6
Nilsson et al, 2014		★	★	★	★	★	★	★	7

Note: A study can be awarded a maximum of one star for each numbered item within the Selection and Outcome categories. A maximum of two stars can be given for Comparability.

Supplementary Table S1: Sensitivity analysis using trim and fill method for *BRCA* mutation status and breast cancer patient survival outcomes

Variable	No of studies added by T&F	HR (95% CI)	I^2, P†
Overall survival			
BRCA1	3	1.50 (1.16 to 1.93)	69.3%,< 0.001
BRCA2	0	1.50 (1.03 to 2.19)	65.4, 0.002
Breast cancer-specific survival			
BRCA1	1	1.08 (0.76 to 1.53)	68.8, 0.0007
BRCA2	0	1.16 (0.82 to 1.66)	50.9, 0.106
Event-free survival			
BRCA1	2	0.96 (0.73 to 1.26)	76.8, < 0.001
BRCA2	2	0.93 (0.66 to 1.30)	44.6, 0.094

Abbreviations: CI, confidence interval; HR, hazard ratio; T&F, trimmed and filled analysis, using assumption of random effects.
